# From field of dreams to back to the future? Exploring barriers to participating in continuing professional development (CPD) programs

**DOI:** 10.1186/s12909-024-05038-5

**Published:** 2024-02-01

**Authors:** Udoka Okpalauwaekwe, Carla Holinaty, Tom Smith-Windsor, James W. Barton, Cathy MacLean

**Affiliations:** 1 Department of Academic Family Medicine, College of Medicine, University of Saskatchewan, Saskatoon, SK S7M 3Y5 Canada; 2 College of Medicine, Victoria Hospital, Prince Albert, SK S6V 5T4 Canada; 3 College of Medicine, University of Saskatchewan, Saskatoon, SK S7N 5E5 Canada

**Keywords:** Faculty engagement, Faculty development, Continuing medical education, Continued professional development, Barriers, Community, Education, Teaching

## Abstract

**Background:**

In 2009, Yvonne Steinert et al., at McGill University, published a study exploring barriers to faculty development (FD) participation among urban faculty. Over a decade later, we set out to replicate and expand on that study to learn what has changed in continued professional development (CPD) and what the current barriers are to participation in CPD for specialists and family physicians in rural and urban locations.

**Methods:**

Informed by a collaborative inquiry research framework, we invited faculty across rural and urban Saskatchewan to focus groups and interview sessions. The results were analyzed for themes.

**Results:**

Thirty-four faculty members from both rural and urban areas participated in this study. Of these, 50% were female, 74% practiced in urban areas, and 56% had over 20 years of experience. Frequently cited reasons for nonparticipation included time constraints, organizational and logistical challenges, poor resonance with material and presenters, and lack of recognition for teaching provided. Racism contributed to feelings of disconnectedness among physician faculty members.

**Conclusion:**

Even after more than a decade, our research uncovered consistent reasons for nonparticipation in locally organized CPD events. New findings highlighted feelings of disconnectedness, notably stemming from racism and workplace discrimination. However, with recent societal developments brought about by the COVID-19 pandemic, can we ride these major waves of change to a new future of engagement? The pandemic led to a shift to virtual and hybrid professional development programs, presenting both benefits and challenges. Additionally, the peri-COVID anti-racism movement may positively address previously unidentified reasons for nonattendance. Harnessing these major changes could lead to a new future of engagement for continued professional development.

**Supplementary Information:**

The online version contains supplementary material available at 10.1186/s12909-024-05038-5.

## Introduction

Continuing professional development (CPD), including faculty development (FD) and continuing medical education (CME), are planned programs designed to provide educational support for faculty members [1, 2].

Accrediting bodies and licensure require physician and faculty involvement in CPD. As such, physicians are primarily engaged in patient care and participate in accredited CPD programs required to maintain licensure [3]. Universities and clinical institutions are mandated as part of their accreditation to offer CPD for their physicians, but a review of recent literature revealed that physicians are disengaged from their institutional CPD programs, such as locally organized FD and/or CME activities, and/or pursue them outside their institutions [3–6]. Faculty engagement in the context of this work refers to the involvement, commitment, and active participation of faculty members in activities that enhance and advance their knowledge, skills, and competencies in teaching, research, and clinical practice [3, 6]. It encompasses participation in learning programs (e.g., seminars, workshops, conferences, etc.), reflective clinical and professional practice, and contribution to scholarly activities in respective fields of clinical practice as of faculty [6].

Several studies and reports have shown that most physicians and faculty are disengaged from their institutional CPD programs, including their locally organized FD and/or CME programs [7–14], and for a number of reasons [13–19]. This was the focus of the Steinert et al., studies [18, 19] which we sought to replicate and expand upon in our research. Common reasons for faculty disengagement in the literature included, lack of time and physician burnout [7–10], content relevance [4], cost and convenience [4, 11, 12], de-incentivized or uncompensated training events [20], misperceptions of the value of these events to their professional practice [21], logistics (program coordination with location, personnel, supplies) [22], institutional dearth of purpose [9, 13, 14], lack of institutional and peer support [4, 15], technological barriers [15], job dissatisfaction [23, 24], dislike for team collaborations [25], fear of losing control [13, 25], and outright indifference to physician engagement events [6, 13].

In Saskatchewan, physician (faculty) engagement remains a well-recognized priority area [26]. A 2021 Saskatchewan health research report concerning physician engagement and leadership actions emphasized similar challenging factors to physician engagement and underscored the necessity for institutional cooperation and fostering a sense of professional unity [27]. A survey of Saskatchewan physicians revealed physician disengagement from local CPD programs, attributed to unfavorable leadership and administrative policies, communication gaps, inadequate management training, limited workforce, and organizational issues [26]. Additionally, consistently low registrations for locally organized CPD programs have been observed among physicians and faculty at the University of Saskatchewan’s College of Medicine, which has over 2,000 faculty members scattered across urban and rural sites throughout the province. The Division of Continuing Medical Education (CME), the Office of Faculty Development (FD), and Distributed Medical Education (DME) aim for more active participation in Saskatchewan-based CPD programs by Saskatchewan physician faculty members. Thus, this study focused on two research questions: (1) What do faculty members identify as barriers to participation in university organized CPD programs, and (2) how can faculty engagement with CPD programs be enhanced?

## Methods

### Context

Saskatchewan is one of Canada’s 13 provinces and territories with a population of over 1 million, half of which live in major cities, Saskatoon, and Regina [28]. The province boasts a distinct population distribution that includes urban, metropolitan, indigenous communities and rural and remote areas [29]. As of March 31, 2020, Saskatchewan had 2,622 licensed physicians, of whom 1,330 were family physicians, with 52% in urban areas, 25% in rural areas and 23% in smaller cities [30]. Most physicians in the province have an academic appointment with the College of Medicine at the University of Saskatchewan.

### Positionality and reflexivity

CM, JB, TSW, and CH are Canadian board-certified practicing physicians with leadership positions in FD, CME and DME in the College of Medicine. UO is a physician and PhD candidate with the University’s College of Medicine and is not affiliated with the University’s FD, CME or DME departments. UO facilitated participant recruitment, data collection, and data management (collection, analysis, and interpretation). The research team met biweekly via videoconference to discuss concerns, resolve conflicts, and ensure strict adherence to research protocols.

### Study design and methods

This study design was guided by a collaborative inquiry framework, which is grounded on the principles of participatory research [31]. Within this participatory framework, and from the participants perspectives, we gain insights into how we might work collaboratively to explore faculty concerns and what barriers exist to enhanced participation. Within this framework, we actively engage participants to learn from their perspectives how we could collaboratively address the concerns of faculty engagement delineated in our study objectives. Our study methods included focus group discussions and interviews.

### Study setting

This study was conducted in the Faculty Development office of the University of Saskatchewan. All focus group discussions and interviews were conducted using Zoom (Zoom Video Communications, San Jose, CA) and Webex (Cisco Systems, Milpitas, CA) platforms. Participants included physicians (MDs) and other academics (i.e., PhDs) irrespective of their attendance at university organized CPD events.

### Participant recruitment and sampling strategy

We employed a mixture of purposive and convenience sampling approaches while estimating that a sample size between 15 and 30 participants would achieve outcome saturation for our study. Invitations were sent from the FD office to all registered physicians and medical faculty members with the University of Saskatchewan via e-mails and word of mouth.

### Ethical considerations

This study was reviewed by the University of Saskatchewan behavioral ethics board and received exemption status as per Article 2.5 of the Tri-Council Policy Statement (TCPS): Ethical Conduct for Research Involving Humans [32].

### Data collection methods and instruments

Informed consent was sought prior to data collection. We conducted three separate focus group discussions for each category of participants (i.e., attendees, non-attendees, and PhDs). Participants who could not join any of the discussion sessions had individual interviews. Each focus group discussion session lasted approximately 60 min, while the interviews lasted between 30 and 45 min. In both methods, we explained the purpose of the study, guiding the discussions using a semi-structured interview guide for consistency (see Supplementary File A for guiding questions for all sessions). The pilot tested interview guide explored the following questions: (a) What discourages you from attending university-organized CPD programs, and (b) What ways do you think can enhance participation in CPD university-organized programs? All sessions were audio recorded, and field notes were taken. All data were stored in the FD office under the supervision of the principal investigator (CM).

### Data processing and analysis

All audio-recorded data were transcribed for thematic analysis following the steps prescribed by Braun and Clarke [33]. Individual transcripts and field notes were analyzed line-by-line and categorized based on interview questions. We used NVivo version 12 (QSR International, Burlington, MA) to code, categorize, and quantify participant responses by frequency and emerging themes. The University’s Canadian Hub for Applied and Social Research (CHASR) also independently analyzed the transcripts for contents and themes. The team determined the study had reached data saturation after non new information emerged nearing the end of the data collection. All themes and subthemes were shared with participants and research team members for feedback and validation of interpretation.

### Techniques used to enhance rigour and trustworthiness

We employed the strategies for ensuring trustworthiness in qualitative research as proposed by Guba [34], Shenton [35], and Patton [36]. Credibility was established through member-checking with participants. Confirmability was ensured through independent data analysis, triangulation and comparing codes and emerging themes for similarities. For validation and clarity, we pilot-tested our interview questions with 5 individuals prior to commencement. Research biases were addressed via researcher reflexivity and debriefing meetings. We achieved transferability by adhering to the Standards for Reporting Qualitative Research [37] for study reporting.

### Findings

#### Demographic information of all participants

We interviewed 34 faculty members. Of all our participants, 32 were physicians, and 2 were PhDs. Seventeen were male, 25 were urban physicians, and nine were rural physicians. Eighteen were family physicians, while other specialists included anesthetists, surgeons, emergency medicine physicians, critical care specialists, internists, and pathologists. The duration of professional practice ranged from 3 years to over 20 years. Seventeen were White. Twenty were identified as non-attendees to CPD programs. Detailed descriptions are presented in Table 1.

**Table 1:** Descriptive characteristics of participants (n = 34)

Descriptive variable	Frequency (%)
**Methods**	
Individual interviews	18 (53.0)
Focus group discussions (3 separate sessions with different participants in attendance)	16 (47.0)
*Frequency of attendance to university organized CPD	
Attendees	14 (41.2)
Non-attendees	20 (58.8)
**Location of Practice	
Urban	25 (73.5)
Rural	9 (26.5)
**Canadian trained or Internationally trained (Physicians only n = 32)**	
Canadian Medical Graduate (CMG)	18 (56.3)
International Medical graduate (IMG)	14 (43.7)
**Sex**	
Male	17 (50.0)
Female	17 (50.0)
**Ethnicity**	
White	17 (50.0)
Black	7 (20.5)
Asian/Middle Eastern	10 (29.4)
Indigenous (First Nation, Metis, Inuit)	0 (0.0)
**Years of practice**	
< 5 years	4 (11.8)
6 to 10 years	5 (14.7)
11 to 19 years	6 (17.6)
≥ 20 years	19 (55.9)
**Years affiliated with the USask**	
< 5 years	9 (26.5)
6 to 10 years	8 (23.5)
11 to 19 years	4 (11.8)
≥ 20 years	13 (38.2)
**Specialties/Divisions**	
Family Medicine	18 (52.9)
Surgery (1ENT, 2 General surgeons, 1 ObGyn)	4 (11.8)
Pathology (1 MD, 2 PhDs)	3 (8.8)
Hematology	2 (5.8)
Anesthesiology	2 (5.8)
Emergency Medicine	2 (5.8)
ICU/Critical care Medicine	2 (5.8)
Psychiatry	1 (2.9)

Key: ENT: Ear, Nose and Throat; ER: Emergency Medicine; ICU; Intensive Care Unit; ObGyn: Obstetrician-Gynecologist; University of Saskatchewan

* Attendees were defined as physicians and faculty who had attended a CPD event within the last two years from the commencement of our study

**Urban describes cities of Saskatoon and Regina (two biggest cities in Saskatchewan with population over 300,000 respectively). Areas outside this are considered rural in this study

### Synthesis and interpretation

**Fig. 1:**
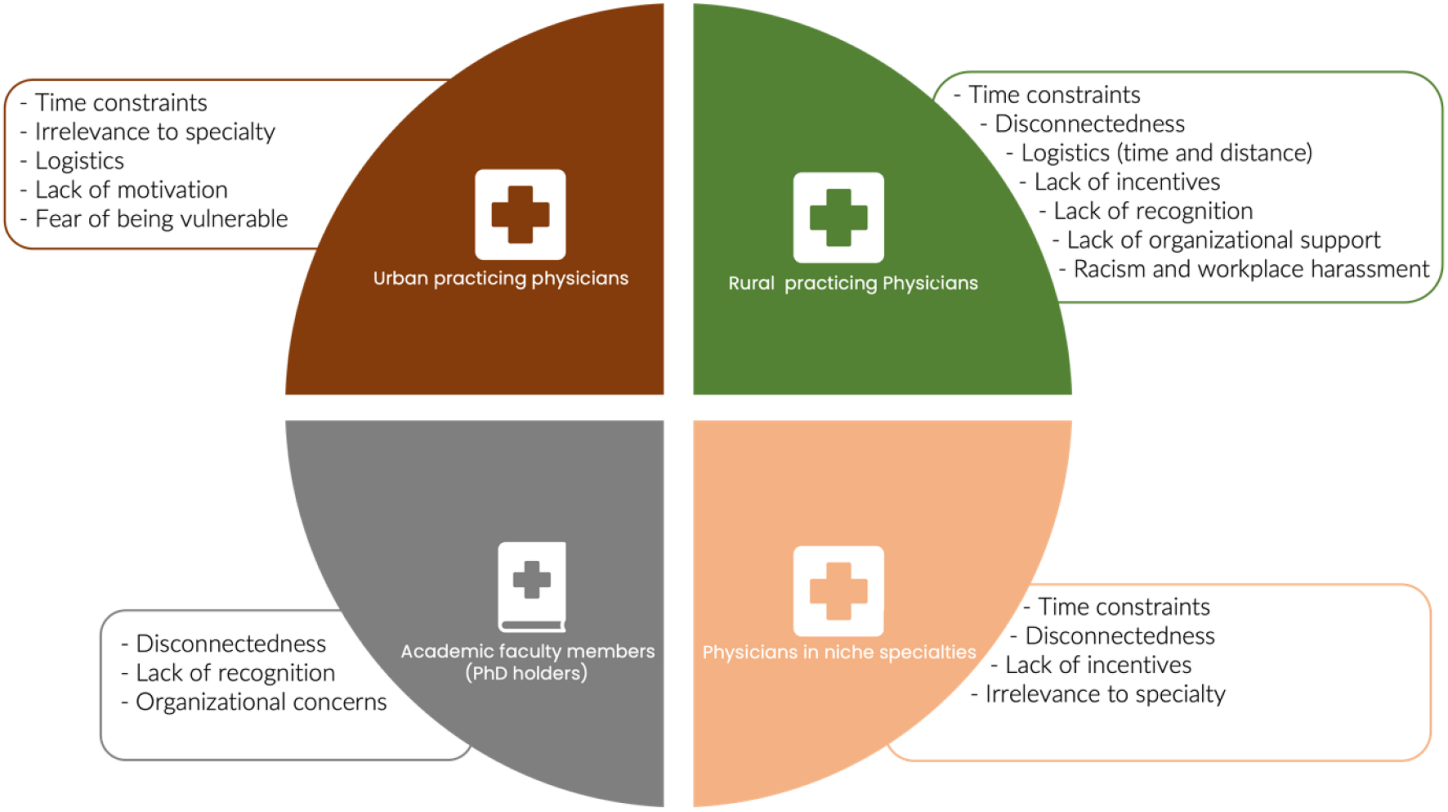
Key barriers to faculty engagement by urban, rural, PhD faculty, and niche specialties (n = 34). Key: Urban practicing physicians, including family physicians and surgeons. Rural practicing physicians include family physicians and specialists working in rural areas. Academic faculty members are PhD degree holders within the College of Medicine. Physicians in niche specialties include specialists in anesthesiology, hematology, pathology, and ENT.*Urban describes the cities of Saskatoon and Regina (the two biggest cities in Saskatchewan with populations over 300,000 respectively). Areas outside this are considered rural in this study

**Fig. 2:**
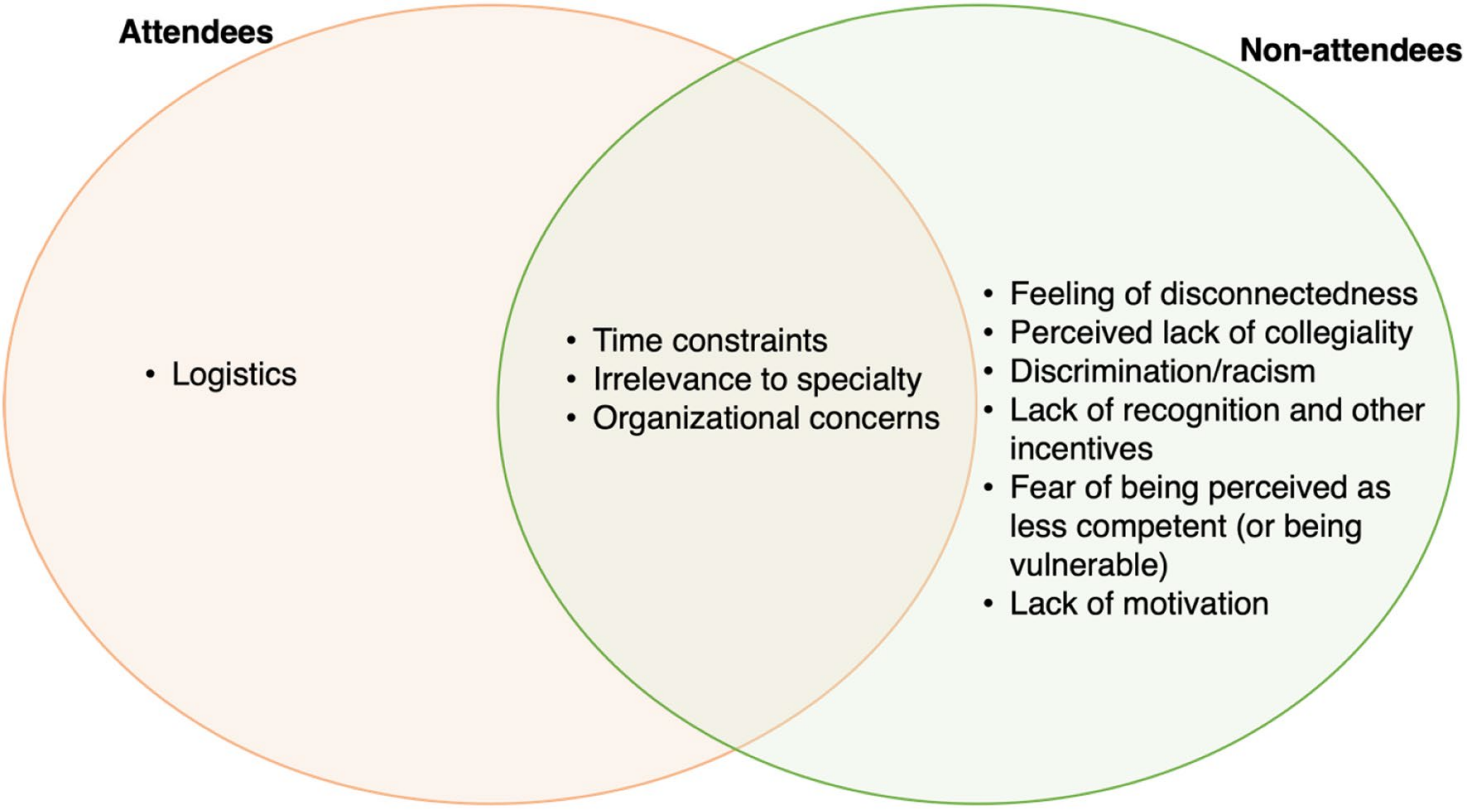
Key barriers to faculty engagement by frequent attendees and non-attendees (*n* = 34). * Attendees were defined as physicians and faculty who had attended a CPD event within the last two years from the commencement of our study

Given the wide range of participants, we used the term ‘CPD’ to refer to both FD and CME unless specified. It is important to highlight that not all our participants had a strong teaching role. Those who were not engaged in much teaching put stronger emphasis on the role of CME (rather than FD) in their affiliation with the university. Figures 1 and 2 provide an overview of the findings from this study categorized for sub-groups (Fig. 1 categorized for urban, rural, specialist and PhDs; Fig. 2 categorized by attendees and non-attendees). For this study, we only report findings to the questions that explored: (a) reasons for non-participation in university-organized CPD programs and (b) ways to enhance participation in CPD university-organized programs. We grouped our findings into the following themes and subthemes:

### What are the barriers to participating in CPD programs?

We noted seven themes on why faculty members did not participate in university organized CPD programs:

#### Time constraints and lack of remuneration models for participation

Time constraints were the most common theme and reason given for nonparticipation in CPD activities. Twenty-one (61.8%) participants reported time constraints due to competing priorities such as clinical work, research, personal time with family, administrative demands, and the impacts of burnout.

There is limited time available for educational activities…I have been given 10% of my time toward teaching and research at the university. 10% is very tiny, but then asking me to attend College of Medicine activities like the FD is an extra ask of my time rather than my employer’s time.

Time constraints were also closely related to faculty members’ contract type/remuneration model. Physicians with the College of Medicine are remunerated in several ways that interplay with their perceived response to physician engagement. Models of remuneration include salaried, contracts and fee-for-service (FFS).

So, I work fee for service, so if I’m not working, I am not earning money.

Trying to convince people to do more than they are paid for is truly a challenge. So… that’s the main impediment to faculty development.

#### Organizational and logistical concerns

Organizational and logical concerns were another common theme (19/34; 55.9%) identified as barriers to participating in CPD programs. These included the relevance and applicability of CPD topics to practice, the perceived academic quality of presenters, modes, and nature of advertisements for CPD programs (e.g., verbose and lengthy e-mails versus concisely worded catchy titles), meeting venue (in-person versus virtual), location (centralized versus scattered), time of the event, travel distance, parking, and the general organizational structure of university-organized CPD programs. Fifteen (15/34; 44.1%) participants cited that the topics advertised for CPD were either too lengthy to read, difficult to sign up for, lacking in catchy phrases that ignite enthusiasm, or showed no relevance to their fields of practice.

I am a family physician in a niche field…I stopped attending because I didn’t find what is discussed relevant to advancing my knowledge in my field….

FD and CME programs don’t have punchy lines or topics that are appealing to my field….

The quality of CPD program delivery also challenged participation. Two urban physicians described their unwillingness to participate in locally organized CPD programs due to the perceived poor quality of program organization and the caliber of presenters invited to these events.

…in my humble opinion, we need to increase the calibre of the delivery of the CME so people can be interested….

I have been here for many years, and I haven’t seen anything change in the type of speakers presenting….

Participants also cited that centralized CPD programs were nearly impossible for faculty members working outside of Saskatoon to attend in person. Additionally, connecting virtually to a CPD event designed to be attended in person was not preferred because of other issues, such as the feeling of exclusion and other technical problems associated with connectivity.

It is overwhelming when all you do is interact with others through a screen…it is even more overwhelming when I am the host….

#### A perceived lack of accountability

Some physicians expressed a feeling of frustration with the lack of accountability, direction and supervision by the university’s leadership structures, describing these as barriers to engaging in CPD programs.

If the College was valuing my teaching, they would actually have somebody come and look at it and then tell me how to do it better.

As far as the university goes, there are no reviews, no quantification of the teaching or effort or learning activities or growth. We don’t [have them], and there are no reviews or anything of what work we do for the university.

Additionally, although the tracking of credits for CME appeared to ease the question of accountability for some, there was still that challenge for FD events causing physicians to prioritize clinical work and its competencies over their academic commitments.

Physicians who identify themselves mostly as clinical physicians, feel that patient care and safety, are more important than teaching….

#### A perceived lack of a sense of community and collegiality among faculty

The lack of community and camaraderie was a recurring theme among most rural physicians (7/9; 77.7%), a few urban physicians in niche specialties (3/25; 12.0%), and the PhD faculty. A newly employed faculty member speaking about the reasons for not engaging in CPD programs commented on the challenges of navigating between work and other faculty-related activities.

It’s a weird position I feel like I’m in, because…nobody knows me when I try to engage with them… not only is there disconnectedness, but it is also like nobody truly cares….

In addition to the lack of community and camaraderie, approximately 30% of physicians spoke about their experiences with racism and discrimination being internationally trained medical professionals and the hostile working environment this created.

…there is racism. In addition, so, coming into a CPD meeting as a person of color, people look like, what are you doing here, and act surprised to see you.

…There is also the distinction between international medical graduates and Canadian medical graduates, which determines how they engage with you….

Two physicians in niche specialties expressed a feeling of disconnection from the College, as most events were tailored to areas outside their specialties.

There’s almost no sense of community in the other people who may be attending…the connectedness to my niche is sometimes overlooked. In addition, so, I go elsewhere for CME….

The PhD faculty members also expressed some level of disconnectedness or exclusion from CPD programs organized within the College of Medicine.

… I fully understand that being part of the College of Medicine… has been to produce MDs as well as support their continuing medical education… but we don’t feel included as part of the College…they don’t feel the need to pass on information with us.

Lastly, with the world engaged in only virtual events over the year of the study, it was even harder for some participants to start or renew a sense of community when all attendees were sitting in their own spaces and unable to communicate in a way that fostered collaboration and community.

This last year has been a lot of virtual retreats, webinars, and that kind of thing. In addition, I truly miss the interaction, the more direct interaction with colleagues. I truly miss the contact. It’s just not quite the same over video.

… it’s harder to engage over a virtual platform casually. I think that is the biggest problem.

#### A perceived lack of recognition and incentives

A few participants (4/34; 11.8%) expressed the absence of recognition and incentives (financial or nonfinancial) for their roles and efforts in the areas of teaching and research as barriers.

Speaking about FD activities, I will be on the borderline of that and say, there’s very little incentive for participation….

…there’s no recognition if we do an extra teaching or involvement or education or other things anyway.

…there’s no remuneration. I was not deducted time in any way. I was allowed to have some time from my employment to attend as a continuing education opportunity; still, there was no financial incentive for doing that.

#### Fear of being vulnerable, and lack of motivation

Three participants described the fear of being vulnerable or being perceived as less competent as their peers as a barrier to participating in university organized CPD programs.

I think some people might find it embarrassing to reveal themselves as perhaps being less capable compared to their peers. I think it’s put some of them in a vulnerable position which can be threatening.

Two participants mentioned a lack of external motivation for personal development regarding teaching as a challenge to CPD attendance.

…we truly struggle with faculty development… there’s truly no motivation…, I guess other than internal motivation, you know, to be a better teacher and a better educator.

### How can faculty engagement in CPD programs be enhanced in the college?

Participants suggested ways faculty engagement could be enhanced to encourage participation. These included:

#### Building stronger communities of practice

All participants cited community building as a critical factor in engagement. A few ways suggested are listed below:

Involving physicians in decision-making processes to ensure programs are designed to match professional needs.

I guess if people were to reach out to us and determine what we’re interested in and maybe what topics would generate some attendance over time.

Building personal and interpersonal relationships among colleagues in the College of Medicine.

…the vast majority are not going to see the benefit until we start building relationships among ourselves.

Promote diversity within CPD program delivery.

…we should find a way to recruit diverse people, for example, someone of Nigerian ancestry comes in, you want to attend;… some Pakistani or (an) Indian person.

#### Building empathy toward physicians to nurture their inherent love for learning

Participants expressed that they are more than simply faculty – they have multiple roles, and often these roles are at odds with one another. Continuing professional development was identified as important to their faculty roles but must not be delivered and collected in a way that is further burdensome or neglectful of the inherent love of learning that most identified within themselves.

So having CME that is flexible, that doesn’t feel like one has to sacrifice time against life-work-balance, and where the love of learning is utilized.

#### Incentivizing participation in CPD activities

This was a common theme among young faculty members with fee-for-service remuneration. While financial incentives were acknowledged, the participants expressed other ways to incentivize CPD programs (such as the caliber of the speaker, the topic of discussion, method of delivery, quality of presentation, acknowledgments, awards, credits, and recognition) to encourage participation.

*“You need to spark interest for the physicians to be partnered. It should be considered as part of not only the requirement but also their achievement. I’d like to see a better incentive… It’s not always about the money; it’s the respect.*

#### Integration of programs and activities delivered to physicians

Physicians feel they receive many e-mails from several affiliated programs under the same organization and other medical organizations. Therefore, integrating multiple programs into a singular platform would be a way to encourage participation.

…I get tonnes of emails from different medical bodies under the University about their own CME… they’re not well integrated. Having them work together would help.

#### Providing more support for medical faculty to thrive in their career pursuits

With the multiple complaints with the lack of accountability for CPD, participants felt that the College could do much more, starting with the hiring process. Participants suggested that the College should attract and retain higher calibre physicians with expectations clearly defined at the time of hiring. They also recommended that CPD events not be add-ons (e.g., tagged into pre-existing meetings) but separate events with engaging speakers that discuss interesting, relevant, experiential, and consensus- chosen topics. Finally, physicians recommended having local support to assist faculty in achieving CPD deliverables and expectations. Supports such as funding, grants, mentorship, and accountability exemplified.

#### Determining areas of interest or need among faculty members

There were advocates among the participants to have CPD topics that were consensus driven. Many participants highlighted the importance of an engaging and relevant topic for CPD. A couple of participants suggested surveying the faculty to determine how to proceed with CPD in the future:

I think that the content should also be consensus driven. What do the majority want to learn and experience?

## Discussion

This study was motivated by the observation of limited physician engagement in CPD activities, which is often a shared experience. Although Yvonne Steinert et al. [18, 19] carried out a similar study in 2008 and 2010, respectively, we wanted to explore the same questions within our local context. The 2008 study by Steinert et al., titled “Faculty Development: a Field of Dreams,” primarily investigated why clinical faculty members did not attend Faculty Development programs [18]. The faculty members in Steinart's studies were based at an urban institution and the study conducted focus groups that were solely composed of clinical faculty members who had not taken part in any such programs [18]. Expanding on Steinert’s work [18, 19], our study delved into the perspectives encompassing both formal and informal CPD programs for faculty situated in rural and urban areas, extending to specialists, family physicians and faculty with PhD qualifications. Additionally, we opened invitations to both regular and non-attendees. Our primary objective was to understand the reasons for the non-attendance to university organized CPD events among faculty. Our study unveiled barriers such as time restrictions, organizational and logistical concerns, lack of accountability for teaching, lack of comradery and community, lack of recognition, fear, and motivational factors. These key findings resonate with findings from several related studies [4, 7, 15] including the studies by Steinert et al. [18, 19].

We believe our CPD programs are largely informed by the needs of the faculty, staff, medical residents, and medical students. The curriculum for these programs which include Faculty Development (FD) and Continuing Medical Education (CME), are conscientiously tailored to the context of the department/division under the College of Medicine. As administrators in CPD, we try to incorporate innovative strategies and respond to feedback provided by our program evaluations and insights from both students and faculty alike. It comes as a surprise that some faculty members believed the topics presented were irrelevant to the needs of participants. Perhaps, there may be a disconnect not only in the content of our programs but how they are communicated and advertised. Steinert’s study [19], using a value-expectation framework showed that individuals were drawn towards activities that align with their expectations and perceived benefits [19]. A huge need for improvement in the area of engaging communication may be warranted. This may require further inquiry and perhaps some marketing and promotional strategies.

Time constraints were the most common reasons for nonparticipation in CPD programs. This comes as no surprise, as most physicians have demanding clinical schedules, compounded by shortages and burnout, which leads to prioritizing clinical work over other activities, including CPD programs [4, 15]. Time constraints may have also been exacerbated, because of the different payment models at the college, which were described by some, as logistical concerns that created a disconnect between the anticipated levels of participation in CPD activities and their contractual obligations. Moreover, the relevance of CPD content to individual practice areas, the perceived quality of program delivery, and other logistical concerns such as location and timing were particularly notable among rural physicians and those in niche specialties. These, to some extent, have been ameliorated using virtual platforms and hybrid formats to encourage participation. However, while virtual platforms have been sought to mitigate this, some participants found them unhelpful and disengaging. Many, especially attendees in our study favored hands-on engagement approaches. Additionally, concerns about virtual learning fatigue and combining in-person and virtual CPD programs were discussed. These concerns open opportunities for future research on optimizing CPD programs for time constraints and enhanced hybrid delivery methods.

Our study unveiled significant workplace discrimination and racism among rural and internationally trained physicians, which hindered participation in CPD programs. This was not reported in the Steinhart et al. study. While not entirely new, these issues require significant attention. A 2019 survey by the Saskatchewan Medical Association (SMA) revealed that 35.2% of physicians encountered or witnessed racial discrimination in the workplace by colleagues and patients [38]. In our study, 50% of participating physicians identified as people of color, mainly Asian or Black. Moreso, international medical graduates (IMGs) shared instances where they were passed over job opportunities, had their medical competencies questioned, were treated unfairly by work colleagues, and were subjected to microaggressions, sometimes by colleagues, but most often by patients. We intend to further explore this issue separately to learn in-depth experiences and mitigating anti-racist strategies that could be adopted to curb these issues within the medical school and the healthcare system in Saskatchewan. The SMA and the College of Medicine are dedicated to fostering equity, diversity, and inclusion among physicians in their commitment to building a healthier medical profession in the province [39].

Our study highlighted the absence of financial incentives or compensation for financial losses due to participation in CPD programs. This further underscores the prevalent prioritization of clinical work, which is closely tied to contract types, and payment models restricting time for non-clinical activities. This clinical imperative makes physicians see themselves as clinicians first, which, although not inaccurate, implies the false perception that CPD is not connected to clinical practice. Research has shown a positive correlational relationship between participating in CPD and physicians’ clinical performance [40, 41], leading to improved patient care [11, 15, 42, 43]. In essence, if physicians could see CPD programs as non-competing with clinical work but rather as facilitating quality care in clinical practice would be a different approach to curbing some of these barriers. Again, promotional and marketing from a professional perspective could help. Additionally, we have come to believe that most physicians did not realize that financial compensation for participating in university organized accredited CPD programs is made available and can be claimed through the provincial medical association. Perhaps increasing awareness and access to compensatory avenues could help as well.

### Lessons learned and practical implications

Our study holds implications for practice and further research. Recognizing that time and logistical concerns are universally faced by physicians, adaptations to promoting CPD programs while accommodating for time restrictions are crucial. Potential adaptations could include sending notices for CPD programs earlier than usual to allow forward planning, sending reminders for CPD programs periodically to give faculty members adequate time to adjust their schedules, and running CPD programs consistently and at certain times to enhance predictability. Additional strategies could include having options for asynchronous CPD programs, integrating micro-FD or CME activities into routine physician meetings, and adopting program marketing with more engaging, pithy titles.

Furthermore, to engage participation catered to the expectations of faculty may involve exploring to understand the expectations and value perceptions of faculty members. This might involve more needs assessment, and further inquiry into what strategies and support could enhance personal development in teaching, research, and learning. Steinert’s studies [18, 19] showed a variety of teaching and learning methods as a facilitator to CPD engagement. The use of more interactive and participatory teaching/learning methods, more personalized content and the assessment of how CPD program benefits are communicated were a few suggestions.

Cultivating a sense of community among faculty members is a gradual process that in large part hinges on building relationships. We had initially created communities of practice in several sites in the college to encourage community building but soon discovered that one can’t force relationships on others who don’t want them. We learned that creating the environment and structures that foster camaraderie tended to flourish more organically rather than through forced interactions. This takes time and patience. Our study revealed racism and workplace discrimination which was lacking in Steinert’s study. We intend to further explore this in future studies and bring racism as a recurring topic of discussion in our CPD events moving forward. As with any other institution, we believe racism in medicine necessitates ongoing discourse and implementation of appropriate changes.

### Future directions and recommendations

Our study findings lay the groundwork for future directions and call for a responsive, faculty-centered approach to CPD in academic medical environments. Future research would evaluate the impacts of integrated and adaptive CPD models, currently set in place, on the varied professional and personal contexts of our faculty members. Additionally, addressing the experiences of discrimination, racism, and workplace harassment by racialized faculty members, in hopes of implementing targeted strategies to foster community and address these crucial issues would be a pertinent step to enhance faculty engagement in the province. Furthermore, raising awareness about the value of locally organized CPD programs is crucial to mitigating these discriminatory barriers and enhancing the CPD experience of participants. Recommendations to facilitate future directions and next steps may include 1) showcasing and celebrating local faculty expertise, 2) establishing departmental recognition for involvement in CPD, 3) paying attention to career transition points (e.g., medical school to residency, residency to clinical practice, clinical practice to academic practice) where young health professionals can appreciate and learn the value of CPD to clinical practice, 4) establishing a mentorship program for younger faculty navigating the challenges of settling in, 5) enlisting marketing experts to promote university-organized CPD activities, and 6) physician engagement in quality improvement.

## Conclusion

This study explored the reasons behind faculty members’ non-participation in CPD programs and how these programs could be better tailored to suit their needs. Overall, our study reinforced similar reasons to those identified by Steinert et al. [18, 19] while uncovering additional issues like racism and workplace discrimination as a barrier to non-participation in CPD programs. Our study unveiled that collaborative partnerships with faculty on these key issues are essential to achieving effective and sustainable reforms within CPD programs transitioning from a field of dreams to a future of hope and advancing engagement.

### Electronic supplementary material

Below is the link to the electronic supplementary material.

Supplementary Material 1: Semi-structured focus-group/interview guide

## Data Availability

The datasets used and/or analyzed during the current study available from the corresponding author on reasonable request.

## Abbreviations

CME: Continuing Medical Education
CPD: Continuing Professional Development
ENT: Ear, Nose and Throat
EM: Emergency Medicine
FD: Faculty Development
ICU: Intensive Care Unit
USask: University of Saskatchewan
