# Supplementary material for: From field of dreams to back to the future? Exploring barriers to participating in continuing professional development (CPD) programs
Source: BMC Med Educ. 2024 Feb 1;24:106. doi: 10.1186/s12909-024-05038-5 (PMC10835933; doi:10.1186/s12909-024-05038-5)
Supplement: Supplementary file 1 — Supplementary Material 1: Semi-structured focus-group/interview guide [file 12909_2024_5038_MOESM1_ESM.docx]

**Supplementary File A**

1. **Focus group discussion and interview guide.**

**Perceptions of FD/CME (or CPD)**

1. What does Faculty Development mean to you? (What comes to mind when you hear the word FD)
2. What does Continuing Medical Education/CME mean to you?

- How do you see faculty development and/or CME relevant to your work as a faculty member?

1. Are you aware of any faculty development programs or events provided by faculty development in your department or the CoM (previously in person or virtual; locally, provincially)?

- If so, how did you become aware of these faculty development activities?
- Similarly, how do you learn about CME activities in the province?

1. We understand the demands on your time and how busy things can get. In light of that, I would like to know if you have attended any CoM FD/CME/CPD activity in the past couple of years. If so, what have you attended (in-person or virtual (industry sponsored); locally, provincially)?

[for those who answer yes, follow up with questions]

- How often did you attend any FD, CME or CPD activity in the last two years?

Once 2-3 times 3-4 times  5-10 times >10 times

- What content areas would be most relevant to you?
  - In FD?
  - In CME?

1. **Barriers and facilitators to participation**
2. What discourages you from attending USask FD or CME activities? Are the reasons different for FD and CME?

You may read the following (in individual sessions):

Studies have shown that common reasons for failing to attend FD or CME activities among physicians include the following:

1. Time restrictions (e.g., protected time, research obligations, clinical care of patients, administration, personal life, etc.)
2. Logistics (e.g., central location of organized activities)
3. Lack of reward (e.g., lack of recognition or financial reward)
4. Lack of direction of FD/CME
5. Disconnectedness to the university (e.g., disconnect of FD/CME activities to university)
6. Insufficient support from institutions (FD or CME)
7. Underestimation of need for FD or CME (e.g., teaching is not for me, advancing learning is irrelevant)

5a. Does any of the above listed resonate with your experience as a faculty member, teacher, physician, or researcher? If not, what other reasons serve as a barrier to attending FD/CME activities? To what extent are they a barrier?

1. What encourages you to attend USask FD or CME activities? Are the reasons different for FD and CME?

You may read the following (in individual sessions):

Studies have also shown that common reasons that encourage physicians to attend FD or CME activities include the following:

1. Social/community benefits (e.g., sense of congeniality and community)
2. Personal benefits (e.g., study credits)
3. Professional benefits (e.g., expectations from colleagues, promotion, addressing local needs)
4. Therapeutic/emotional benefits

6a. Does any of the above listed resonate with your experience as a faculty member, teacher, physician, or researcher? If not, what other reasons motivate you to attend FD/CME activities and to what extent?

1. Aside from the burden of time, what professional and/or other obligations may challenge attendance to these activities?

Probe for responses on (in focus group discussions):

- - Clinical care of patients
  - Research/Quality improvement or other faculty obligations
  - Other work obligations
  - Administration/committees
  - Personal life
  - Other?

1. What are some reasons you would consider that could pose barriers to participation in FD or CME programs?

Probe for responses on (in focus group discussions):

1. Time (protected)
2. Logistics (central location, organization of activities)
3. Lack of reward (recognition, financial reward)
4. Lack of direction of FD/CME
5. Disconnectedness to the university (disconnection of FD/CME activities to the university)
6. Insufficient support from institutions (FD or CME)
7. Underestimation of need for FD or CME (teaching is not for me, advancing learning is irrelevant)
8. What would motivate you to attend (a session elsewhere or centrally)?
   - How does getting virtual access to events help? (are they different for FD and CME?)
9. **Ways to increase FD/CME participation as prescribed by previous studies**
10. What strategies have you found helpful to move your career along and improve your practice as a physician/specialist?
11. (Following up on the above) In what ways can the FD/CME provide that support to facilitate career advancement and/or self-improvement?

- What works now?
- What is not working now?
- How have things changed since March 2020

1. **Preferred teaching and learning methods**
2. What are your favorite ways to learn? (case studies, small groups, lectures, experience learning, self-directed, simulations, exams and questionnaires)

- Does FD/CME activities align with these preferred learning methods?
- What educational formats would be of most interest to you? (Lectures? Workshops? Short sessions? Webinars? Podcasts?)
- Most of the FD/CME activities have moved online since March 2020. Are you satisfied with this current option? (Based on other responses, people find it inundating, impersonal and sometimes distracting). We want to be sure we are meeting your needs.
- How does it influence your desire to participate and why?
- (Depending on response from above), probe to get a response on what can be done by the FD and CME to mitigate that?
- Some FD/CME is during the day and some is in the evening? How does timing during the day influence your decision to participate?
